# Group-Aware Registration for Lesion-Level Quantitative Motion Correction in Respiratory-Gated PET/CT Biomedical Imaging

**DOI:** 10.3390/s26144554

**Published:** 2026-07-17

**Authors:** Hui Zhou, Longxi He, Yangsheng Hu, Zhouyuan Qin, Feng Wang, Jianfeng He

**Affiliations:** 1 Faculty of Information Engineering and Automation, Kunming University of Science and Technology, Kunming 650500, China; 2 College of Engineering, Yuxi Normal University, Yuxi 653100, China; 3 Key Laboratory of Carcinogenesis and Translational Research (Ministry of Education), Beijing Key Laboratory of Research, Investigation and Evaluation of Radiopharmaceuticals, NMPA Key Laboratory for Research and Evaluation of Radiopharmaceuticals (National Medical Products Administration), Department of Nuclear Medicine, Peking University Cancer Hospital & Institute, Beijing 100142, China

**Keywords:** respiratory-gated PET/CT, respiratory motion correction, deformable image registration, quantitative PET imaging, standardized uptake value, lesion spatial correspondence

## Abstract

Respiratory-gated PET/CT generates phase-resolved biomedical imaging data, but respiratory motion can cause spatial mismatch and lesion-level quantitative instability, especially for small thoracoabdominal lesions. This study proposes SCAR-Net, a Similarity-Constrained Adaptive Respiratory Registration Network, for retrospective phase-to-reference correction of respiratory-gated ^18^F-FDG PET/CT. SCAR-Net treats motion correction as a quantitative stability problem in phase-resolved PET/CT rather than only a generic registration task. It combines sampled group-aware feature encoding with adaptive group-attentive modulation to represent structured respiratory deformation and enhance motion-sensitive correspondence. The method was evaluated using controlled respiratory simulations and a retrospective two-center clinical cohort of 100 patients, jointly assessing lesion-level SUV repeatability, spatial correspondence, image similarity, deformation plausibility, center-stratified performance with B-spline FFD as a conventional reference, cross-dataset testing without target-domain fine-tuning, ablation behavior, and computational efficiency. In the independent clinical test set, lesion-level evaluation included 20 patients, 43 independent PET-avid lesions, and 245 evaluable lesion-phase pairs. In small-lesion phase pairs, SCAR-Net reduced median phase-to-reference variability to 6.45% for |ΔSUV_max_| and 4.73% for |ΔSUV_mean_|, and increased median lesion Dice from 0.55 to 0.72. In large-lesion phase pairs, SCAR-Net achieved a post-correction Dice of 0.89 and remained competitive for SUV repeatability. Descriptive center-stratified analysis showed a consistent lesion-level performance pattern across the two clinical acquisition settings. These findings suggest that SCAR-Net can improve phase consistency and quantitative stability in respiratory-gated PET/CT, with the clearest benefit observed in small-lesion assessment. Downstream clinical endpoints, such as diagnostic accuracy, tumor staging, PERCIST-based response assessment, and patient outcomes, were not evaluated and require future prospective validation.

## 1. Introduction

Respiratory-gated positron emission tomography/computed tomography (PET/CT) provides phase-resolved quantitative imaging data for thoracoabdominal oncology by separating radiotracer uptake into different respiratory states rather than relying on a single time-averaged acquisition [1,2]. This acquisition strategy reduces motion blurring and preserves information about respiratory-phase-dependent lesion position and uptake. However, the gated image series also introduces a signal-consistency problem: each respiratory phase is reconstructed from fewer counts and may contain residual motion, attenuation-correction mismatch, and phase-dependent anatomical displacement. As a result, phase-specific PET/CT measurements may remain spatially inconsistent and quantitatively unstable even after gating.

This instability is most evident when focal lesions are small or located near motion-sensitive anatomical boundaries, such as the diaphragm or lung base. In these regions, small residual displacements can change lesion overlap, apparent uptake distribution, and standardized uptake value (SUV) measurements through a combination of respiratory motion and partial-volume effects [3,4]. Motion correction in this setting should therefore improve not only visual alignment, but also the consistency of phase-resolved PET/CT measurements. A correction method that improves global image appearance may still leave local lesion-adjacent mismatch unresolved, thereby limiting the reliability of lesion-level quantitative assessment.

Conventional deformable registration and respiratory motion-correction methods provide an important foundation for retrospective PET/CT alignment [5]. Variational, diffeomorphic, and model-based approaches can impose smoothness and deformation regularity, but they often depend on iterative optimization, careful parameter tuning, and acquisition-specific assumptions [6,7,8]. Learning-based registration methods reduce the computational burden by predicting deformation fields directly from image pairs, making them attractive for retrospective gated PET/CT workflows [5,9,10,11,12,13]. Yet many of these methods are still optimized or judged mainly through whole-image similarity metrics. Such metrics are useful for assessing global alignment, but they may not fully reflect whether phase-to-reference correction improves lesion correspondence, local signal consistency, or quantitative stability in PET/CT. Therefore, a clinically oriented evaluation should include lesion-level SUV repeatability and spatial correspondence, together with conventional image-level and deformation-based metrics.

A key difficulty is that respiratory deformation in thoracoabdominal PET/CT is structured but not spatially uniform. Motion near the diaphragm and lower lungs may involve coupled displacement, orientation-dependent boundary movement, local expansion or contraction, and region-dependent deformation amplitude [14,15,16,17]. Meanwhile, PET lesion signals are often blurred, noisy, and weakly contrasted against surrounding tissue. These properties make purely voxel-wise matching fragile in lesion-adjacent and motion-sensitive regions. They also suggest that respiratory motion correction may benefit from a representation that captures structured deformation patterns while retaining the flexibility to refine local correspondence.

To address this problem, we developed SCAR-Net, a Similarity-Constrained Adaptive Respiratory Registration Network for phase-to-reference motion correction in respiratory-gated ^18^F-FDG PET/CT. SCAR-Net uses sampled group-aware feature encoding to represent structured respiratory deformation patterns and adaptive group-attentive modulation to refine motion-sensitive correspondence cues within a multi-scale deformable registration framework. Rather than treating registration only as a global image-matching task, this study focuses on whether phase-to-reference correction can improve lesion-level measurement consistency and quantitative stability while maintaining plausible deformation behavior. The evaluation was designed to test this objective across controlled simulations and a retrospective two-center clinical cohort, with particular attention to lesion-size-dependent performance and center-stratified clinical behavior. Thus, the novelty of this work lies in the integration of a respiratory-motion-oriented registration architecture with lesion-level quantitative evaluation for phase-resolved PET/CT, rather than in a single architectural component alone.

The main contributions of this study are as follows:

(1) From an architectural perspective, SCAR-Net combines sampled group-aware feature encoding with adaptive group-attentive modulation to support motion-sensitive phase-to-reference correction in respiratory-gated PET/CT.
(2) From a task-formulation perspective, motion-correction performance is examined from the perspective of phase consistency and quantitative stability, combining lesion-centered SUV repeatability and spatial correspondence with supportive analyses of global image similarity and deformation plausibility.
(3) From an evaluation perspective, the evaluation includes controlled respiratory simulations and a retrospective two-center clinical cohort, with learning-based baseline comparisons, a conventional B-spline FFD reference in center-stratified clinical analysis, lesion-size subgroup analysis, cross-dataset testing without target-domain fine-tuning, ablation experiments, and inference-time assessment.

## 2. Related Work

### 2.1. Respiratory Motion Correction in PET/CT and Gated Imaging

Respiratory motion correction has long been studied in thoracoabdominal PET/CT because breathing can affect lesion localization, attenuation correction, and quantitative uptake measurement. Respiratory gating reduces motion blurring by reconstructing PET data into separate respiratory phases and is useful for selected mobile lesions, especially those close to the diaphragm or lung base [1,2]. However, gated reconstruction also reduces count statistics within each phase and cannot by itself ensure that lesion position and uptake remain consistent across the respiratory cycle. Registration-based correction is therefore commonly used after gated reconstruction to improve phase correspondence and reduce residual motion effects.

Conventional respiratory correction strategies, including deformable registration, motion modeling, and diffeomorphic optimization, provide explicit regularization and interpretable deformation control [1,18,19]. These properties are valuable in PET/CT, where anatomically plausible motion fields are required. However, such methods often rely on iterative optimization, careful initialization, and parameter tuning. Their performance can be sensitive to PET-specific factors such as low spatial resolution, low-count noise, heterogeneous tracer uptake, attenuation-correction mismatch, and protocol differences across scanners or gating schemes. These limitations have motivated more efficient image-pair registration methods for repeated phase-to-reference correction.

### 2.2. Deformable Image Registration and Deep Learning-Based Registration

Deformable image registration has been widely used for anatomical alignment, multimodal fusion, radiation therapy guidance, and image-guided navigation. Classical registration frameworks usually optimize an image-similarity term together with deformation regularization. Recent medical-imaging studies also show that registration accuracy is most informative when interpreted in relation to the downstream task, such as target registration error, anatomical overlap, learned multimodal similarity, or navigation accuracy [20,21,22]. In quantitative PET/CT, this task-dependent perspective is important because improved global alignment does not necessarily imply stable lesion-adjacent signal or phase-consistent quantitative measurement.

Learning-based registration reduces the computational cost of iterative optimization by predicting deformation fields directly from image pairs. In medical image analysis, U-Net-type encoder–decoder architectures have become a common design paradigm because they combine multi-scale contextual encoding with spatial detail recovery through skip connections. A recent analytics-driven review of U-Net for medical image segmentation summarized the development of U-Net variants and highlighted the importance of encoder–decoder design, skip connections, and multi-scale feature fusion in medical imaging [23]. Although segmentation and registration have different outputs, these architectural principles have also influenced many dense prediction networks used for deformable registration. VoxelMorph is a representative unsupervised CNN-based framework for dense deformation prediction and has become a common baseline in medical image registration [24]. Later models introduced richer contextual modeling and stronger moving–fixed interaction. TransMorph combines convolutional and Transformer components to capture long-range volumetric correspondence [25], while XMorpher uses cross-attention between moving and fixed images to strengthen paired-image feature interaction [26]. These methods improve the efficiency and flexibility of deformable registration, making them attractive for respiratory-gated PET/CT workflows in which multiple phases must be aligned to a reference phase.

Despite these advances, most general registration networks are not designed around the specific signal characteristics of respiratory-gated PET/CT. PET phases are often low-count, blurred, and intensity-heterogeneous, and lesion signals may be weak relative to surrounding uptake. A model optimized mainly for whole-image similarity may therefore improve global appearance while leaving local motion-sensitive correspondence insufficiently corrected. This limitation is particularly relevant when correction performance is expected to support quantitative stability rather than only visual alignment.

### 2.3. Geometry-Aware and Group-Aware Representation for Motion-Sensitive Imaging

The limitations of generic image-pair registration have encouraged methods that represent deformation with more structured motion priors. Thoracoabdominal respiratory motion is not a uniform displacement field; it may include coupled organ displacement, local expansion or contraction, orientation-dependent boundary movement, and region-dependent motion amplitude. Motion-decomposition and grouped-representation methods are therefore relevant to respiratory imaging. ModeT formulates deformable registration through motion decomposition and represents multiple motion modes within a Transformer-based framework [27]. GroupMorph combines grouping-based deformation estimation with contextual fusion to capture deformation information across different receptive-field ranges [28]. These approaches suggest that structured representations can be helpful when motion is spatially complex and locally variable. However, their use in respiratory-gated PET/CT remains challenging because the data are phase-resolved, low-count, and lesion-sensitive.

## 3. Materials and Methods

### 3.1. Study Design and Data Preparation

This study evaluated phase-to-reference respiratory motion correction in respiratory-gated ^18^F-FDG PET/CT using controlled simulation datasets and a retrospective two-center clinical cohort. The simulated datasets were generated using GATE v8.0 [29] and included 120 cylindrical phantom cases (CyDat) and 60 cardiopulmonary voxel phantom cases (CaDat). Both datasets modeled a 5 s respiratory cycle with diaphragm displacement ranging from 1 to 3 cm. Simulated PET volumes were zero-mean-normalized, intensity-scaled to [0, 1], and cropped to 128 × 128 × 32. Data were divided at the case level into training, validation, and testing subsets using a 6:2:2 ratio, corresponding to 72/24/24 cases for CyDat and 36/12/12 cases for CaDat.

The clinical cohort included 100 respiratory-gated ^18^F-FDG PET/CT studies collected from the Peking University Cancer Hospital and The First People’s Hospital of Yunnan Province. Assuming one gated PET/CT study per patient, this cohort corresponded to 100 patients, including 56 patients from Beijing and 44 patients from Kunming. Across the full clinical cohort, 279 independent PET-avid measurable lesions were identified, yielding 1642 evaluable lesion-phase pairs after ROI adjudication. In this study, independent lesions referred to distinct patient-level lesions, whereas lesion-phase pairs referred to phase-specific observations of these lesions across non-reference respiratory phases. The two centers used different scanner platforms, acquisition modes, and respiratory-gating protocols, providing a heterogeneous setting for phase-to-reference correction (Table 1). For each study, the end-expiratory phase was used as the fixed reference phase, and all remaining respiratory phases were treated as moving phases. For network input and deformation-field estimation, clinical PET volumes were intensity-normalized and resampled to 64 × 96 × 96. The original attenuation-corrected SUV-calibrated PET volumes were retained for lesion-level quantitative endpoint computation, so that SUV_max_ and SUV_mean_ were not derived from normalized network-input images. The clinical cohort was divided at the patient level into training, validation, and testing subsets using a 6:2:2 ratio, corresponding to 60/20/20 studies. This patient-level split prevented phase-level leakage between training and testing.

Lesion regions of interest (ROIs) were delineated on attenuation-corrected PET/CT images for lesion-level spatial and quantitative evaluation. Two nuclear medicine physicians independently delineated PET-avid measurable lesions at each respiratory phase using PET/CT fusion for localization and CT guidance when lesion margins were visible. In this study, the registration network operated on gated PET phase images; CT information was used for attenuation correction, anatomical localization, and ROI adjudication, but CT volumes were not used as direct network inputs. Discrepant contours were reviewed according to predefined ROI quality criteria and adjudicated when necessary. Lesion-phase pairs that remained non-evaluable after adjudication were excluded from lesion-level endpoint computation. Inter-reader agreement for lesion ROI delineation was quantified using the Dice similarity coefficient [31], with the results across all evaluable clinical lesion-phase pairs in the full clinical cohort reported in Table 2.

For lesion-level quantitative evaluation, the deformation field estimated from the normalized PET input pair was applied to the corresponding SUV-calibrated moving PET volume and mapped it onto the reference-phase grid. PET intensity images were warped using linear interpolation, whereas binary lesion masks were warped using nearest-neighbor interpolation. ${SUV}_{max}$ and ${SUV}_{mean}$ were computed within the adjudicated reference-phase lesion ROI on both the fixed reference PET volume and the corrected moving PET volume. This common-ROI strategy was used to evaluate phase-to-reference quantitative consistency after spatial correction. Absolute phase-to-reference SUV variability was calculated as the percentage difference between the corrected moving phase and the fixed reference phase. For lesion-size subgroup analysis, each evaluable lesion-phase pair was assigned to the small- and large-lesion subgroup according to the diameter of the corresponding independent lesion, using a 10 mm threshold.

### 3.2. Phase-to-Reference Registration Formulation

The motion-correction task was formulated as within-study three-dimensional phase-to-reference deformable registration. For each gated PET/CT study, let $I_{f}:\;\Omega \to R$ denote the fixed end-expiratory reference volume and $I_{m}:\;\Omega \to R$ denote a moving non-reference respiratory phase, where $\Omega \subset R^{3}$ is the image domain. The registration network predicts a dense displacement field $u:\;\Omega \to R^{3}$, which defines the deformation mapping ϕ(x)=x+u(x) [32,33]. The corrected moving image is obtained by warping the moving phase to the reference grid [34]:

(1) $${\hat{I}}_{m}(x)=I_{m}(\phi (x))$$

The same predicted displacement field was used for image-level and lesion-level evaluation. PET intensity images were warped using linear interpolation, whereas binary lesion masks were warped using nearest-neighbor interpolation for lesion spatial correspondence and ROI transfer. Thus, image similarity, deformation plausibility, lesion correspondence, and SUV-based measurements were computed from the same phase-to-reference deformation field.

### 3.3. SCAR-Net Architecture

**Figure 1:**
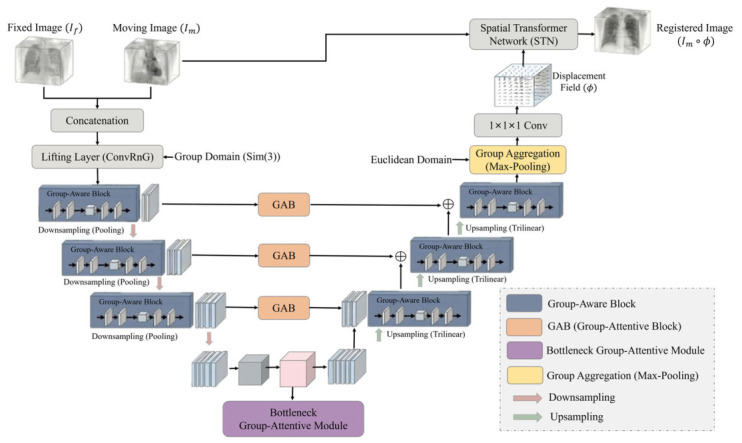
Overview of SCAR-Net for phase-to-reference respiratory motion correction in gated PET/CT. The concatenated moving and fixed PET volumes are encoded into a sampled group-indexed feature space, refined by Group-Attentive Blocks, decoded into a Euclidean feature representation, and regressed into a dense displacement field for spatial-transformer-based warping.

SCAR-Net is a multi-scale encoder–decoder network for phase-to-reference deformable registration in respiratory-gated PET/CT. The moving and fixed PET volumes are concatenated as a two-channel input, and the network outputs a dense three-dimensional displacement field. The architecture contains four encoding levels and four decoding levels. As shown in Figure 1, the concatenated image pair is first lifted by an initial 3 × 3 × 3 convolution, after which sampled group-aware convolutional blocks apply predefined Sim(3)-related transformations to the intermediate feature representation and extract transformation-indexed responses according to the selected group size G. These group-indexed features are further refined through adaptive group-attentive modulation and then decoded for displacement regression. The final displacement field is generated by a 1 × 1 × 1 convolution and applied to the moving phase through a spatial transformer.

**Figure 2:**
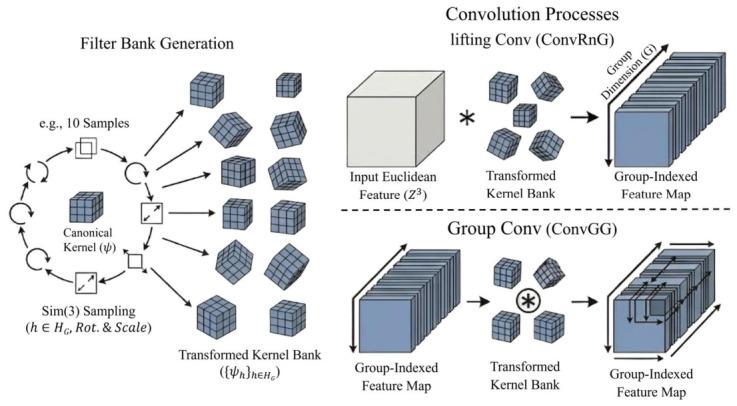
Sampled group-aware feature encoding for structured respiratory deformation. A canonical 3D kernel is transformed by a finite set of sampled spatial operations to generate a transformation-indexed filter bank. The transformed kernels are used for lifting convolution from Euclidean features to transformation-indexed features and for subsequent group-aware convolution. In the main SCAR-Net configuration, G = 32 sampled transformation responses were generated by combining eight representative SO(3) rotation elements with four scale factors sampled from [0.8, 1.2]. The reduced G = 8 setting used the eight rotation elements only and served as an ablation configuration. Therefore, G controls the number of sampled transformation responses retained by the encoder. The asterisk (*) denotes the corresponding convolution operation, namely lifting convolution in the upper branch and group-aware convolution in the lower branch.

To represent structured respiratory deformation, SCAR-Net uses sampled group-aware feature encoding (Figure 2). In this study, the sampled transformation set was implemented as a finite Sim(3)-inspired approximation rather than a full continuous Sim(3)-equivariant representation. Let [35]

(2) $$H_{G}={\left\{h_{g}\right\}}_{g=1}^{G}$$

denote a retained set of sampled transformation elements, where *G* is the number of transformation-indexed responses used by the encoder. Each $h_{g}$ defines a predefined spatial operation used to transform the convolutional kernel. In the reduced G = 8 setting, the sampled set consisted of eight representative orientation-preserving rotations from SO(3), with adjacent sampled rotations separated by 45° in the adopted discrete orientation parameterization. In the full G = 32 setting, these eight rotations were combined with four scale factors sampled from [0.8, 1.2], yielding 32 rotation-scale transformation elements.

Given a canonical 3D kernel k, the transformed kernel associated with $h_{g}$ is written as [36,37]:

(3) $$k_{h_{g}}(\xi )=k({S_{h_{g}}}^{-1}\xi )$$

where ξ denotes the local kernel coordinate and $S_{h_{g}}$ denotes the linear spatial component associated with the sampled transformation element $h_{g}$. This component represents the sampled rotation operation in the G = 8 setting and the sampled rotation-scale operation in the G = 32 setting. The transformed kernels form a sampled filter bank, which is used for lifting convolution from Euclidean features to transformation-indexed features and for subsequent group-aware convolution, as illustrated in Figure 2. Therefore, G denotes the number of retained sampled transformation responses, not the order of the full continuous Sim(3) group.

In the implementation, the transformation-indexed responses were concatenated along the channel dimension to form a group-expanded feature representation. Group normalization was applied after group-aware convolution, followed by LeakyReLU activation with a negative slope of 0.2. When resampling was required during the sampled transformation operation, trilinear interpolation was used to match the target spatial size before feature fusion. This finite sampling strategy introduces transformation-aware feature responses while keeping three-dimensional PET registration computationally tractable. The main SCAR-Net configuration used G = 32 sampled elements, whereas G = 8 was examined in the ablation study.

**Figure 3:**
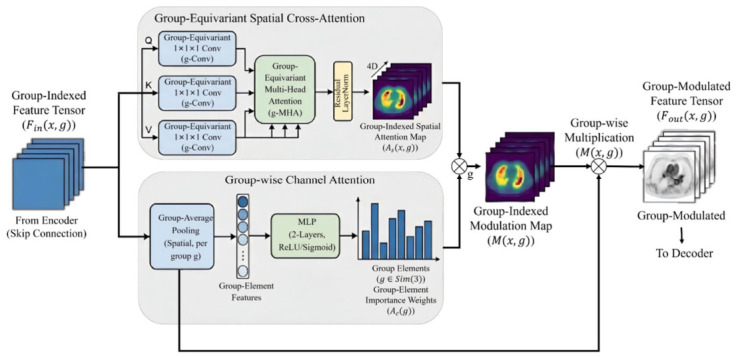
Group-Attentive Block for adaptive feature refinement. The block combines group-aware spatial attention with group-wise channel weighting to recalibrate group-indexed features before decoder fusion.

Adaptive Group-Attentive Blocks (GABs) are inserted into the skip pathways and bottleneck to refine group-indexed features before decoder fusion (Figure 3). Let $F_{l}(x,c,h)$ denote the group-indexed feature tensor at encoding level l, spatial location *x*, channel *c*, and sampled transformation element $h\in H_{G}$. Each GAB contains a group-aware spatial attention branch and a group-wise channel attention branch. The spatial branch generates a transformation-indexed spatial modulation map $A_{s}^{l}(x,h)$, which emphasizes motion-sensitive regions in the feature space. The channel branch estimates group-wise channel weights $A_{c}^{l}(c,h)$, which recalibrate transformation-indexed feature responses before decoder fusion. The combined modulation weight is defined as:

(4) $$M_{l}(x,c,h)=A_{s}^{l}(x,h)A_{c}^{l}(c,h)$$

and the refined feature is

(5) $$F_{l}^{GAB}(x,c,h)=M_{l}(x,c,h)F_{l}(x,c,h)$$

This formulation summarizes the role of GAB: spatial attention enhances local motion-sensitive correspondence cues, whereas channel attention adjusts the relative importance of transformation-indexed responses. The refined group-indexed features are then passed to the decoder through skip connections. After decoder fusion, the decoded group-indexed feature tensor is converted into a Euclidean feature representation before displacement regression. This group aggregation is written as:

(6) $$F_{E}(x,c)=\underset{h\in H_{G}}{\max}F_{D}(x,c,h)$$

where $F_{D}$ denotes the decoded group-indexed feature tensor. The dense displacement field is then regressed by a 1 × 1 × 1 convolution,

(7) $$u(x)=W_{u}*F_{E}(x)+b_{u}$$

and applied to the moving image through the spatial transformer described in Section 3.2. No lesion masks, organ labels, or anatomical segmentations are used as network inputs during registration.

In the implemented network, the encoder used base channel widths of [4,8], and the decoder used base channel widths of [4,8]. These values denote the base channels before group expansion. In sampled group-aware convolutional layers, the effective channel dimension is multiplied by the sampled group size G. Group normalization was used in group-aware convolutional layers, LeakyReLU was used as the activation function, and no dropout was applied.

### 3.4. Training Objective and Implementation

SCAR-Net was trained without ground-truth deformation fields by optimizing image similarity and deformation smoothness. For each fixed-moving PET pair, let $I_{f}$ denote the fixed reference image, $I_{m}$ the moving image, ${\hat{I}}_{m}$ the warped moving image obtained using the phase-to-reference deformation defined in Section 3.2, and u the predicted displacement field. The training objective was defined as:

(8) $$L(I_{f},\;I_{m},\;u)\;=\;L_{\mathrm{NCC}}(I_{f},\;{\hat{I}}_{m})\;+\;\lambda L_{\mathrm{smooth}}(u)$$

where λ=0.01. Local normalized cross-correlation (NCC) [38] was used as the image-similarity term:

(9) $$L_{NCC}\left(I_{f},\;{\hat{I}}_{m}\right)=-\frac{1}{\left|\Omega \right|}\sum_{x\in \Omega }\frac{[{\sum_{p\in N(x)}(I_{f}(p)-\bar{I_{f}}(x))({\hat{I}}_{m}(p)-\bar{{\hat{I}}_{m}}(x))]}^{2}}{\sum_{p\in N(x)}{\left(I_{f}(p)-\bar{I_{f}}(x)\right)}^{2}\sum_{p\in N(x)}{\left({\hat{I}}_{m}(p)-\bar{{\hat{I}}_{m}}(x)\right)}^{2}+\epsilon }$$

Here, Ω denotes the image domain, N(x) is a local 9 × 9 × 9 window centered at x, $\bar{I_{f}}(x)$ and $\bar{{\hat{I}}_{m}}(x)$ are local mean intensities within this window, and $\epsilon =1\times 10^{-5}$. Deformation smoothness was encouraged using a diffusion regularizer:

(10) $$L_{\mathrm{smooth}}\left(u\right)=\frac{1}{\left|\Omega \right|}\sum_{x\in \Omega }\sum_{d\in \{x,y,z\}}{\left\|\nabla u_{d}\left(x\right)\right\|}_{2}^{2}$$

Lesion masks, anatomical labels, and SUV endpoints were not included in the loss function. They were reserved for post-registration evaluation so that the lesion-level endpoints remained independent of model optimization. This design kept training annotation-free and based only on the fixed-moving PET image pair and deformation regularity, which is consistent with routine phase-resolved PET/CT settings where manual lesion contours are not always available for model training.

All models were implemented in PyTorch using Python 3.9 and trained on a workstation equipped with an Intel Core i9-10900K CPU, 32 GB RAM, and an NVIDIA RTX 3080 Ti GPU with 12 GB memory. For SCAR-Net, training used the Adam optimizer with a learning rate of 0.001 and a batch size of 1. The model was trained for 7500 iterations. No early-stopping rule was applied; the checkpoint with the lowest validation loss was selected for testing. Inference time was measured for model prediction and warping on clinical PET volumes and did not include preprocessing.

### 3.5. Baselines, Evaluation Metrics, and Statistical Analysis

Five learning-based registration baselines were selected and retrained for the same phase-to-reference PET/CT registration task. VoxelMorph (VM) was used as a CNN-based unsupervised dense registration baseline [24]. TransMorph (TM) represented Transformer-based global-context modeling [25], and XMorpher (XM) represented cross-attention-based moving–fixed correspondence learning [26]. ModeT was included because motion decomposition is relevant to respiratory deformation [27], while GroupMorph [28] served as a close comparator for grouped deformation estimation and contextual fusion. All learning-based baselines used the same training, validation, and testing splits as SCAR-Net and were trained for 7500 iterations. Because the baseline architectures differ in their original implementations, optimizer-specific settings were retained or adapted as appropriate for each method. The evaluation pipeline, including preprocessing, spatial resampling, intensity normalization, mask-warping rules, and endpoint definitions, was kept identical across all methods.

To provide a conventional non-learning registration reference, B-spline free-form deformation (FFD) registration was additionally evaluated in the center-stratified clinical lesion-level analysis on the independent test set. The FFD baseline was implemented using SimpleITK. For each fixed–moving phase pair, the fixed and moving PET volumes were min–max normalized to [0, 1] for transformation estimation. A B-spline transform was initialized on the fixed-image domain with a mesh size of 4 × 4 × 4. Registration was optimized using a correlation similarity metric with 30% random metric sampling and a three-level multi-resolution scheme with shrink factors of [1, 2, 4] and smoothing sigmas of [2, 1, 0]. Transform parameters were estimated using the L-BFGS-B optimizer, with a gradient convergence tolerance of 1 × 10^−5^, a maximum of 100 iterations, five maximum corrections, 500 maximum function evaluations, and a cost-function convergence factor of 1 × 10^7^. Because the fixed and moving phases were from the same respiratory-gated PET/CT study and were reconstructed on a common image grid, no additional rigid or affine pre-registration was performed. The same FFD parameter setting was applied to all test cases without case-specific tuning. The resulting FFD transforms were evaluated using the same SUV-calibrated PET volumes, lesion masks, interpolation rules, and endpoint definitions as the learning-based methods.

Evaluation was organized around phase consistency, lesion correspondence, global alignment, deformation plausibility, robustness, and computational feasibility. Lesion spatial correspondence was quantified using the Dice similarity coefficient between the warped moving-phase lesion ROI and the adjudicated reference-phase ROI. Lesion-level quantitative stability was assessed using absolute phase-to-reference percentage differences in ${SUV}_{max}$ and ${SUV}_{mean}$. Supportive image-level metrics included the structural similarity (SSIM) [39], peak signal-to-noise ratio (PSNR) [39], normalized mutual information (NMI) [40], and correlation coefficient (CC) [41]. Deformation behavior was assessed using HD95 and the fraction of voxels with non-positive Jacobian determinant where applicable [42]. Robustness analyses and inference-time measurements were used to characterize performance under heterogeneous respiratory and acquisition conditions.

Clinical lesion-level analyses were performed only on the independent test set. This test set included 20 patients, 43 independent PET-avid lesions, and 245 evaluable lesion-phase pairs. Lesion-size subgroup analysis and center-stratified analysis were performed on the same lesion-phase-pair set using different stratification factors. No lesion mask, lesion-phase observation, or SUV endpoint from test patients was used during training, validation, checkpoint selection, hyperparameter selection, or model selection.

Continuous variables were summarized as median and interquartile range unless otherwise specified. Because lesion-level PET measurements were not normally distributed and multiple lesion-phase observations could arise from the same patient, lesion-wise metrics were complemented by patient-level analyses. For paired comparisons, lesion-level measurements were first summarized within each patient using the median, and Wilcoxon signed-rank testing was then performed across patients with Holm correction for multiple comparisons. Bootstrap 95% confidence intervals for selected lesion-level effects were estimated using patient-level cluster bootstrap with 1000 resampling replicates. Statistical analyses were performed in Python using SciPy and related libraries.

## 4. Results

The evaluation was organized to determine whether phase-to-reference correction improved quantitative consistency in respiratory-gated PET/CT while preserving plausible deformation behavior. Therefore, lesion-level SUV repeatability and spatial correspondence were first analyzed as the main task-related outcomes. Global image consistency, deformation plausibility, cross-dataset testing, computational efficiency, and ablation results were then used to further characterize registration behavior, robustness, and practical feasibility.

### 4.1. Lesion-Level Quantitative Stability and Spatial Correspondence

**Figure 4:**
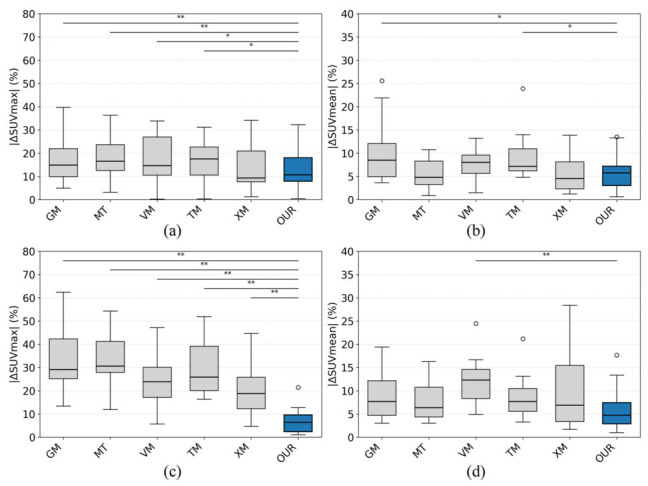
Distribution of lesion-level SUV variability after motion correction. (**a**) absolute phase-to-reference percentage differences in SUV_max_ for large lesions (≥10 mm); (**b**) absolute phase-to-reference percentage differences in SUV_mean_ for large lesions (≥10 mm); (**c**) absolute phase-to-reference percentage differences in SUV_max_ for small lesions (<10 mm); and (**d**) absolute phase-to-reference percentage differences in SUV_mean_ for small lesions (<10 mm). Asterisks indicate paired comparisons with SCAR-Net after Holm correction (* *p* < 0.05; ** *p* < 0.01). The statistical comparisons were based on within-patient summarized lesion-level measurements, whereas the boxplots display the distribution across evaluable lesion-phase pairs.

Lesion-level quantitative stability and spatial correspondence were evaluated on the independent clinical test set. The test set included 20 patients with 43 independent PET-avid lesions, yielding 245 evaluable lesion-phase pairs across non-reference respiratory phases. For lesion-size subgroup analysis, these lesion-phase pairs were stratified according to the diameter of the corresponding independent lesion, resulting in 167 small-lesion phase pairs (<10 mm) and 78 large-lesion phase pairs (≥10 mm). Absolute phase-to-reference differences in SUV_max_ and SUV_mean_ were summarized as median values with interquartile ranges and analyzed separately by lesion size (Table 3; Figure 4). The uncorrected moving phases were included in Table 3 as a descriptive pre-correction baseline to show the magnitude of phase-to-reference SUV variability before deformation-based correction. Patient-level paired comparisons were performed after within-patient summarization and Holm correction, as described in Section 3.5. Corrected significance levels are indicated in Figure 4, whereas the bootstrap confidence intervals in Table 3 provide the corresponding effect-direction information for the median differences relative to SCAR-Net.

In the small-lesion subgroup, the uncorrected moving phases showed median $\left|∆{SUV}_{max}\right|$ and $\left|∆{SUV}_{mean}\right|$ values of 25.93% and 5.58%, respectively. After SCAR-Net correction, these values decreased to 6.45% and 4.73%, indicating reduced small-lesion SUV variability relative to the uncorrected moving phases. The closest competing method for small-lesion $\left|∆{SUV}_{max}\right|$ was XMorpher, with a median value of 18.76%. For small-lesion $\left|∆{SUV}_{mean}\right|$, ModeT and XMorpher were the closest selected baselines, with median values of 6.34% and 6.90%, respectively. Patient-level bootstrap analyses were consistent with these differences, as all baseline-to-SCAR-Net confidence intervals in the small-lesion subgroup were above zero for both SUV endpoints. Together with the Holm-corrected paired comparisons shown in Figure 4, these results indicate that the small-lesion improvement was not only numerically large but also directionally consistent across patients.

In the large-lesion subgroup, the uncorrected moving phases showed median $\left|∆{SUV}_{max}\right|$ and median $\left|∆{SUV}_{mean}\right|$ value of 16.54% and 5.14%, respectively. After SCAR-Net correction, the corresponding values were 10.71% and 3.43%. In this subgroup, baseline variability was lower and between-method differences were smaller. XMorpher achieved the lowest median $\left|∆{SUV}_{max}\right|$ in this subgroup (9.33%), whereas SCAR-Net achieved the lowest median $\left|∆{SUV}_{mean}\right|$. Some bootstrap confidence intervals in the large-lesion subgroup crossed zero, indicating that the separation from the strongest baselines was less consistent than in small lesions. Therefore, the large-lesion results were interpreted as competitive rather than uniformly superior across all SUV endpoints.

Lesion spatial correspondence was assessed by comparing warped moving-phase lesion masks with adjudicated reference-phase ROIs (Table 4). In the small-lesion subgroup, median Dice increased from 0.55 before correction to 0.72 after SCAR-Net correction, corresponding to a median change of 0.17 (95% CI, 0.06 to 0.35). XMorpher and TransMorph were the strongest selected baselines in this subgroup, with corrected Dice values of 0.67 and 0.65, respectively. In the large-lesion subgroup, SCAR-Net increased median Dice from 0.71 to 0.89, with a median change of 0.18 (95% CI, 0.09 to 0.38). XMorpher and VoxelMorph were the strongest selected baselines in this subgroup, with corrected Dice values of 0.85 and 0.82, respectively.

Taken together, these lesion-level findings indicate that SCAR-Net improved phase-to-reference lesion consistency, with the clearest benefit observed in small lesions. The reduction in SUV variability relative to the uncorrected moving phases, together with the increase in lesion Dice, suggests that the correction improved both quantitative repeatability and spatial correspondence. The effect was more pronounced in small lesions, where respiratory displacement and partial-volume effects are more likely to amplify phase-dependent SUV variability, whereas the large-lesion subgroup showed a more competitive pattern with smaller between-method separation.

### 4.2. Center-Stratified Clinical Lesion-Level Analysis

A secondary center-stratified analysis was performed on the independent clinical test set to examine whether the lesion-level performance pattern was consistent across the two acquisition settings (Table 5). The same 245 evaluable lesion-phase pairs were stratified by center. The Beijing subset included 12 patients, 24 independent PET-avid lesions, and 169 lesion-phase pairs, whereas the Kunming subset included eight patients, 19 independent PET-avid lesions, and 76 lesion-phase pairs. B-spline FFD was included as a representative conventional deformable registration reference.

SCAR-Net showed the lowest median $\left|∆{SUV}_{max}\right|$, $\left|∆{SUV}_{mean}\right|$, and the highest corrected Dice in both centers. In the Beijing subset, SCAR-Net achieved $|∆{SUV}_{max}|=8.24\%$, $|∆{SUV}_{mean}|=4.56\%$ and corrected Dice = 0.76. In the Kunming subset, the corresponding values were 7.04%, 3.89%, and 0.80. B-spline FFD improved lesion correspondence in the Beijing subset but showed higher SUV variability than SCAR-Net in both centers. These findings support the center-stratified consistency of the overall lesion-level results, while the limited size of each center-specific subset should be considered when interpreting this analysis.

### 4.3. Image Consistency and Deformation Plausibility

**Figure 5:**
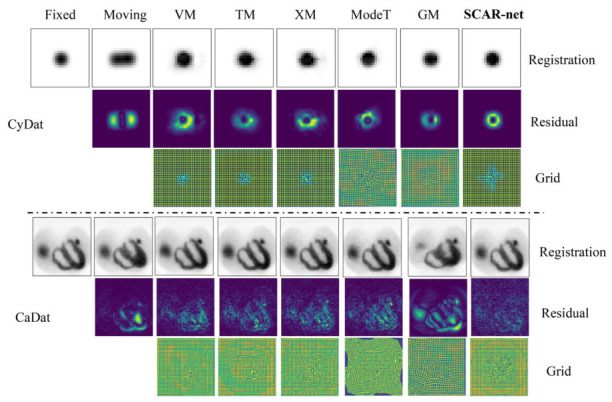
Qualitative registration results on simulated respiratory phantoms. Representative CyDat and CaDat examples show fixed, moving, and corrected images, residual maps, and deformation grids. Lower residual intensity around the moving object or thoracoabdominal boundary indicates improved local correspondence, whereas visually smooth and continuous deformation grids suggest plausible deformation behavior without obvious folding or local distortion.

**Figure 6:**
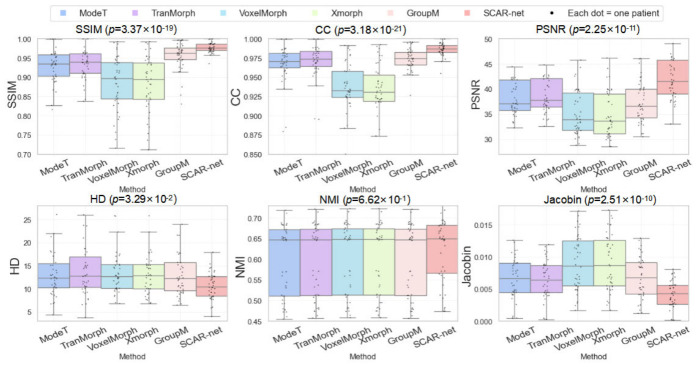
Patient-level supportive image and deformation metrics in the clinical cohort. Boxplots with paired patient-level points summarize SSIM, CC, PSNR, HD95, NMI, and Jacobian-based deformation behavior across methods. SSIM, CC, PSNR, and NMI reflect global image consistency, whereas HD95 and Jacobian-based measurements provide supportive information on residual spatial discrepancy and deformation plausibility.

**Figure 7:**
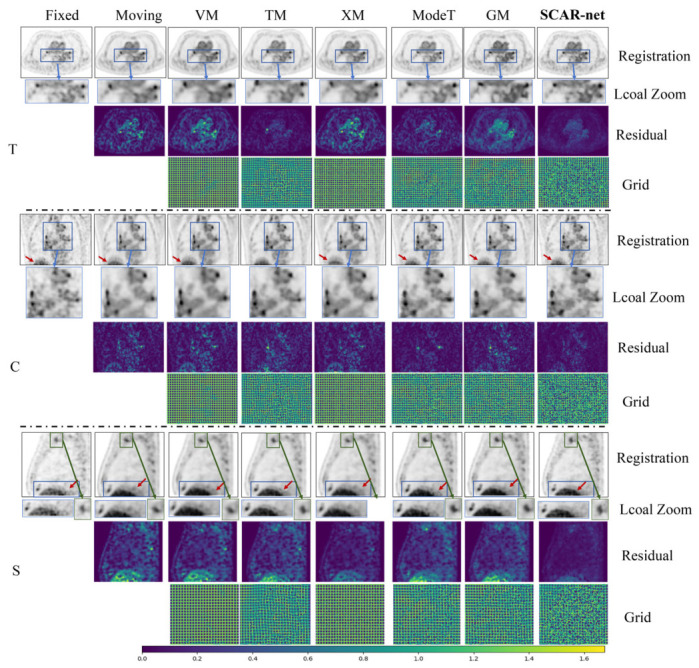
Representative clinical registration examples in transverse (T), coronal (C), and sagittal (S) views. Fixed, moving, corrected, residual, and deformation-grid panels are shown with magnified lesion-adjacent regions. Red arrows indicate representative regions affected by respiratory motion artifacts. The residual maps illustrate changes in local mismatch around the lesion and nearby motion-sensitive anatomical boundaries after correction. The deformation-grid panels provide a visual assessment of spatial smoothness and anatomical plausibility of the estimated deformation.

Following the lesion-level analysis, global image metrics and deformation-related measures were evaluated to determine whether improved lesion consistency was accompanied by stable image alignment and plausible deformation behavior. Controlled simulation phantoms were used to assess registration behavior under predefined respiratory motion conditions, whereas the two-center clinical cohort was used to examine performance under heterogeneous acquisition and respiratory patterns (Table 6; Figure 5, Figure 6 and Figure 7).

In the geometric phantom (CyDat), SCAR-Net achieved the highest SSIM (96.66%) and PSNR (34.17 dB), while maintaining low HD95 (1.29 mm) and a low non-positive Jacobian fraction (0.0039). GroupMorph achieved the lowest HD95 in this phantom (1.02 mm). In the cardiopulmonary voxel phantom (CaDat), SCAR-Net achieved the highest SSIM (96.78%) and CC (99.23%), the lowest HD95 (11.45 mm), and a low non-positive Jacobian fraction (0.00024). These results indicate that SCAR-Net maintained competitive image consistency and deformation plausibility in controlled respiratory simulations.

Qualitative phantom examples showed reduced residual mismatch and visually regular deformation grids after correction (Figure 5). In clinical test cases, patient-wise supportive metrics further showed favorable SSIM, CC, and PSNR distributions, together with low Jacobian-based deformation irregularity (Figure 6). Representative clinical examples highlighted lesion-adjacent regions where residual mismatch is most relevant for quantitative interpretation. Across the displayed cases, SCAR-Net reduced local residual mismatch while preserving visually smooth deformation grids (Figure 7).

### 4.4. Cross-Dataset Robustness Without Fine-Tuning

Cross-dataset robustness was examined by direct testing on a target dataset without additional fine-tuning (Table 7). In this analysis, A denotes CyDat, B denotes CaDat, and C denotes the clinical dataset. A-to-B indicates testing on CaDat using the best checkpoint trained on CyDat. A-to-C and B-to-C indicate testing on the clinical dataset using the best checkpoints trained on CyDat and CaDat, respectively. Source and target datasets did not overlap, and no target-domain fine-tuning was performed.

In the A-to-B setting, SCAR-Net achieved the highest SSIM (94.29%) and the lowest non-positive Jacobian fraction (0.0037), with HD95 comparable to the strongest baselines. In the clinical target settings, SCAR-Net also maintained high SSIM values of 93.09% for A-to-C and 94.09% for B-to-C, together with very low non-positive Jacobian fractions of 0.0001 in both settings. SCAR-Net did not achieve the lowest HD95 in all clinical transfer settings, indicating that direct transfer performance varied across metric categories. Global image consistency and deformation regularity were relatively preserved after direct transfer, whereas HD95 remained more variable in the clinical target settings.

For clinical target testing, small-lesion SUV variability showed a favorable pattern after SCAR-Net correction. In A-to-C, SCAR-Net achieved $\left|∆{SUV}_{max}\right|$ of 4.01% and $\left|∆{SUV}_{mean}\right|$ of 8.04%. In B-to-C, SCAR-Net yielded $\left|∆{SUV}_{max}\right|$ of 2.35% and $\left|∆{SUV}_{mean}\right|$ of 8.69%. These values were among the lowest across methods in both clinical target settings, suggesting that SCAR-Net retained useful lesion-level quantitative behavior during direct testing on the clinical target dataset. Nevertheless, because boundary discrepancy varied across source–target pairs, the cross-dataset results should be interpreted as supportive evidence of robustness under direct transfer, rather than as evidence of uniformly superior generalization across all metrics.

### 4.5. Computational Efficiency and Retrospective Feasibility

Computational efficiency was evaluated using GPU memory consumption, number of trainable parameters, and inference time on CyDat, CaDat, and clinical PET volumes (Table 8). Inference time was reported per moving-reference phase pair and did not include preprocessing.

SCAR-Net required 784,265 trainable parameters, which was lower than ModeT, TransMorph, XMorpher, and GroupMorph, but higher than VoxelMorph. Its GPU memory consumption was 989.79 MB, comparable to TransMorph and lower than ModeT, but higher than VoxelMorph, XMorpher, and GroupMorph. On clinical PET volumes, SCAR-Net required 2002.64 ms per moving-reference phase pair. This was faster than ModeT, TransMorph, and XMorpher, but slower than VoxelMorph and GroupMorph. Overall, these results place SCAR-Net in a practical middle range for retrospective gated PET/CT correction, where offline per-phase processing time is acceptable and lesion-level correction performance is the primary consideration.

### 4.6. Ablation Analysis

Ablation analysis was performed on CyDat, CaDat, and the clinical dataset to characterize the contribution of sampled group-aware encoding, GAB, and group size to image consistency and deformation behavior (Table 9). The evaluated settings included the backbone encoder–decoder, the backbone with sampled group-aware encoding, and SCAR-Net with G = 8 or G = 32. These experiments were used to examine component-level effects and metric trade-offs across configurations. Because the lesion-level results showed the clearest benefit in small lesions, an additional small-lesion ablation analysis was performed on the independent clinical test set using SUV variability and lesion Dice as clinically oriented endpoints (Table 10).

Across all three datasets, adding sampled group-aware encoding improved image consistency relative to the backbone. SSIM increased from 73.68% to 89.20% on CyDat, from 91.57% to 95.30% on CaDat, and from 88.65% to 93.21% on the clinical dataset. The effect on deformation-related metrics was more dataset-dependent. HD95 improved on CyDat and CaDat but increased on the clinical dataset, while the non-positive Jacobian fraction decreased in the clinical setting. These results indicate that group-aware encoding strengthened global alignment but did not by itself optimize all aspects of deformation behavior.

Adding GAB further improved the overall image-consistency profile. SCAR-Net with G = 32 achieved the highest SSIM and PSNR on CyDat, the highest SSIM and CC on CaDat, and the highest SSIM, CC, NMI, and PSNR on the clinical dataset. The CaDat results also showed a marked reduction in the non-positive Jacobian fraction, from 0.0081 in the group-aware-only model to 0.00024 in SCAR-Net with G = 32. In the clinical dataset, SCAR-Net with G = 32 improved image-similarity metrics relative to the group-aware-only model, although HD95 and *J* ≤ 0 did not uniformly favor this configuration. This pattern suggests that GAB improved feature refinement and image consistency, while deformation regularity still depended on dataset characteristics and group size.

The comparison between G = 8 and G = 32 further showed a trade-off between representation capacity and deformation regularity. The G = 32 configuration consistently improved image-similarity metrics, especially SSIM, CC, and PSNR. However, deformation-related metrics were not uniformly better with the larger group size. In the clinical dataset, G = 8 produced slightly lower HD95 and a lower non-positive Jacobian fraction than G = 32, whereas G = 32 provided stronger global image consistency. Therefore, G = 32 was used as the main configuration because it provided the strongest overall image-consistency profile, while the ablation results indicate that group size remains a trade-off rather than a universally optimal setting.

The small-lesion ablation analysis showed that the component-level improvements were also reflected in lesion-level endpoints. The backbone-only model did not improve lesion correspondence in small lesions, with corrected Dice decreasing from 0.55 to 0.48. Adding sampled group-aware encoding reduced $\left|∆{SUV}_{max}\right|$ from 23.91% to 17.29% and $\left|∆{SUV}_{mean}\right|$ from 12.29% to 8.46%, while increasing corrected Dice to 0.63. SCAR-Net with G = 8 further improved these endpoints. The G = 32 configuration achieved the lowest SUV variability and highest corrected Dice, with $|∆{SUV}_{max}|=6.45\%$, $|∆{SUV}_{mean}|=4.73\%$, and Dice = 0.72. These findings indicate that the sampled group-aware representation and adaptive feature refinement contributed not only to global image consistency but also to small-lesion quantitative stability and spatial correspondence.

## 5. Discussion

Respiratory-gated PET/CT provides phase-resolved information, but its value for quantitative assessment depends on whether phase-to-phase differences can be corrected without compromising measurement reliability or introducing implausible deformation. The main finding of this study is that SCAR-Net improved phase-consistent lesion assessment, with the most evident benefit observed in small lesions. This observation is important because small thoracoabdominal lesions are particularly susceptible to respiratory displacement, partial-volume effects, and low PET contrast, making conventional whole-image similarity measures insufficient to fully reflect the reliability of motion correction.

### 5.1. Principal Findings and Lesion-Level Relevance

The lesion-size-stratified analysis showed that SCAR-Net was most effective in the setting where respiratory correction is expected to have the greatest quantitative impact. In small-lesion phase pairs, the method reduced phase-to-reference SUV variability and improved lesion spatial correspondence after correction. The simultaneous improvement in SUV repeatability and lesion Dice is clinically relevant because respiratory motion correction for PET/CT should support phase-consistent lesion assessment rather than maximize a single global similarity score. In this context, the joint improvement in lesion-level quantitative repeatability and spatial correspondence provides direct evidence of the correction effect, particularly for small lesions where respiratory displacement and partial-volume effects are most likely to affect PET interpretation.

The large-lesion results provide a more nuanced interpretation. SCAR-Net achieved strong lesion overlap and favorable SUV_mean_ repeatability, although it was not uniformly the best method for SUV_max_ variability in this subgroup. This is not unexpected. Larger lesions generally have greater baseline spatial overlap and are less sensitive to small residual displacements than sub-centimeter lesions. SUV_max_ is also more sensitive to local voxel-level intensity variation than SUV_mean_, particularly in lesions with heterogeneous PET uptake. These findings suggest that the value of respiratory motion correction is lesion-size dependent, and that small lesions may represent the most informative subgroup for assessing whether registration improves quantitative PET reliability.

The center-stratified analysis further supports this lesion-level interpretation. SCAR-Net showed the lowest median SUV variability and the highest corrected lesion Dice in both clinical centers, despite differences in scanner platform, acquisition protocol, and gating strategy. B-spline FFD, included as a conventional deformable registration reference, showed some improvement in lesion correspondence but retained higher SUV variability than SCAR-Net in both centers. These findings do not constitute independent external validation, because the center-specific subsets were limited in size and were derived from the same retrospective study. Nevertheless, they provide additional evidence that the main lesion-level performance pattern was not driven by a single acquisition setting.

### 5.2. Methodological Interpretation and Deformation Behavior

The performance pattern supports the rationale of combining sampled group-aware feature encoding with adaptive group-attentive modulation. Respiratory deformation in thoracoabdominal PET/CT is not a purely translational process; it includes local displacement, boundary sliding, and phase-dependent changes near the diaphragm, lung base, mediastinum, liver dome, and lesion-adjacent tissues. By introducing sampled transformation-aware feature encoding into an encoder–decoder registration framework, SCAR-Net was designed to provide a structured representation of these deformation patterns before dense displacement regression. The adaptive group-attentive modulation further refines multi-scale features so that correspondence cues in motion-sensitive regions can be emphasized during registration.

The ablation results indicate that these components contributed differently to the final behavior of the model. Group-aware encoding improved image consistency, but did not automatically optimize every spatial discrepancy or deformation-related metric. Adding attention modulation improved the overall image-consistency profile, particularly in the full configuration. The additional small-lesion ablation analysis further showed that these component-level changes were reflected in clinically oriented endpoints. The backbone-only model did not improve small-lesion Dice, whereas adding sampled group-aware encoding and then the full SCAR-Net configuration progressively reduced SUV variability and improved lesion correspondence. This finding links the architectural design not only to global image similarity, but also to the small-lesion endpoint that is most vulnerable to respiratory motion.

The results also show that increasing group capacity is not equivalent to uniformly improving all registration properties. The G = 32 configuration produced stronger image-similarity performance and the best small-lesion SUV and Dice endpoints, whereas the G = 8 configuration showed advantages in some deformation-related measures. This trade-off suggests that sampled group capacity should be considered as a model-design parameter rather than as a simple monotonic improvement factor. For quantitative PET/CT applications, the preferred configuration should be selected according to the intended balance between image consistency, lesion-level stability, deformation regularity, and computational cost.

It is also important that lesion masks, anatomical labels, and SUV endpoints were not used as training supervision. The model was optimized using image similarity and deformation smoothness, while lesion-level endpoints were reserved for independent evaluation. This design avoids direct dependence on manual lesion annotations during training and allows the method to be applied to gated PET phase images without requiring lesion-specific supervision. Future task-specific extensions, such as lesion-aware or SUV-consistency losses, may be worth investigating when larger annotated datasets become available.

### 5.3. Robustness and Retrospective Feasibility

The cross-dataset experiments provide initial evidence that SCAR-Net retained useful registration behavior under domain shifts between simulated and clinical data. This is an important requirement for respiratory-gated PET/CT because scanner platforms, reconstruction protocols, count statistics, gating strategies, and respiratory patterns can vary substantially across institutions. SCAR-Net maintained high-image-consistency values and low-non-positive Jacobian fractions after direct transfer without target-domain fine-tuning. However, HD95 was not uniformly lowest in the clinical target settings, indicating that boundary-level discrepancy remained more sensitive to domain shift than global image similarity or Jacobian-based deformation regularity.

This metric-dependent behavior should be interpreted carefully. HD95 is sensitive to local boundary outliers and may be affected by low-count PET noise, lesion-adjacent uptake heterogeneity, and residual mismatch in anatomically complex regions. In contrast, SSIM, CC, SUV variability, and Jacobian-based metrics summarize different aspects of registration behavior. These findings indicate that the cross-dataset results should not be interpreted from a single metric alone. Rather, direct-transfer performance appeared relatively stable for global image consistency and deformation regularity, whereas local boundary agreement remained more variable across source–target settings. Taken together, the cross-dataset results should be viewed as supportive evidence of robustness under direct transfer rather than proof of uniformly superior generalization across all metrics. This interpretation is consistent with the broader evaluation strategy of this study, in which lesion-level SUV repeatability, lesion Dice, image similarity, residual maps, deformation grids, and Jacobian-related measures were considered together.

From a practical perspective, SCAR-Net showed feasible retrospective processing performance. Its inference time was longer than the lightest convolutional baselines, but shorter than several more complex registration architectures. This places the method in a practical middle range for offline gated PET/CT correction, where per-phase processing time is usually less restrictive than in real-time imaging applications. The current implementation is therefore more appropriately positioned as a retrospective quantitative correction tool rather than as an online reconstruction component. Further engineering optimization may reduce runtime and memory demand, particularly if larger group configurations or higher-resolution clinical volumes are used.

### 5.4. Limitations and Future Work

Several limitations should be considered. First, the clinical evaluation was retrospective and included a moderate-sized two-center cohort. Although the two centers introduced heterogeneity in scanner type and gating protocol, the center-stratified analysis remained descriptive because each center-specific test subset was limited in size. Independent validation across additional institutions, reconstruction settings, lesion locations, and respiratory patterns is still required. The current results support the feasibility and potential value of SCAR-Net for phase-consistent PET/CT assessment, but they do not establish improved downstream clinical outcomes. In particular, this study did not evaluate whether improved phase-to-reference consistency would alter diagnostic accuracy, tumor staging, PERCIST-based response classification, treatment decision-making, or patient outcome. These endpoints require dedicated prospective clinical validation.

Second, lesion-level evaluation depended on expert ROI delineation across respiratory phases. Discrepant contours were adjudicated, and inter-reader agreement was high, but gated PET remains a low-count and low-contrast imaging setting. Boundary uncertainty may vary with lesion size, anatomical location, uptake heterogeneity, and respiratory phase. Therefore, the reported SUV and Dice endpoints should be interpreted as measures of inter-phase consistency within the current annotation protocol, rather than as direct evidence of improved diagnostic classification, treatment response assessment, or survival prediction.

Third, SCAR-Net performs phase-to-reference registration and does not explicitly model temporal consistency over the entire respiratory cycle. This may be relevant in patients with irregular breathing, unstable respiratory amplitude, or phase-sorting errors. In addition, although the main simulation and clinical experiments included residual-map and deformation-grid visualization, the cross-dataset analysis was primarily quantitative. A more complete assessment of cross-dataset transfer would benefit from systematic qualitative review of warped images, residual maps, deformation grids, and lesion-adjacent regions across source–target settings. Such analysis would be particularly useful for interpreting local boundary behavior in cases where HD95 remains sensitive to domain shift. Future work should investigate temporally consistent registration, uncertainty-aware deformation estimation, standardized quality-control criteria, cross-dataset qualitative deformation assessment, and prospective validation in task-specific clinical applications such as radiomics stability, therapy response evaluation, and motion-corrected lesion quantification.

Finally, the conventional registration comparison was limited to B-spline FFD in the center-stratified lesion-level analysis. Although this provided a representative iterative deformable registration reference, it does not cover the full range of classical respiratory motion-correction approaches, parameter-tuning strategies, or hybrid registration pipelines. Future studies should compare SCAR-Net with additional conventional and hybrid approaches under standardized PET/CT-specific evaluation protocols.

## 6. Conclusions

This study introduced SCAR-Net, a group-aware deformable registration framework for retrospective phase-to-reference motion correction in respiratory-gated ^18^F-FDG PET/CT. By combining sampled group-aware feature encoding with adaptive group-attentive modulation, SCAR-Net improved lesion-level SUV repeatability and spatial correspondence, with the clearest benefit observed in small-lesion phase pairs. Center-stratified analysis and comparison with B-spline FFD further supported the consistency of the lesion-level performance pattern across the two clinical acquisition settings. Supportive image-level, deformation, cross-dataset, efficiency, and ablation analyses showed that the method achieved a practical balance between quantitative correction, deformation plausibility, and retrospective feasibility. These findings support the value of lesion-level evaluation as a complement to conventional whole-image metrics and suggest that SCAR-Net may improve phase-consistent quantitative assessment in respiratory-gated PET/CT. However, this study did not evaluate downstream clinical endpoints, including diagnostic accuracy, tumor staging, PERCIST-based treatment response assessment, clinical decision-making, or patient outcomes. Further validation in larger, independent, and prospective cohorts is warranted to determine whether improved phase consistency translates into clinically meaningful benefits.

## Figures and Tables

**Table 1:** Clinical PET/CT acquisition and reconstruction protocols for the two-center cohort. Reproduced from Ref. [30] with permission from Elsevier. Copyright © 2026 Elsevier B.V.

Parameter	Beijing	Kunming
Institution	Peking University Cancer Hospital	The First People’s Hospital of Yunnan Province
Scanner model	United Imaging uEXPLORER	Philips Ingenuity TF PET/CT
PET acquisition mode	Whole-body dynamic PET/CT	TOF-PET/CT with bed-by-bed scanning
Acquisition setting	List-mode, 5 min per bed	Standard gated acquisition, 4 min × 2 beds
Radiotracer and uptake time	^18^F-FDG, 3.7–5.4 MBq/kg, 60 min rest	^18^F-FDG, ~0.15 mCi/kg, 40 min rest
Gating protocol	8 phases, amplitude-based	5 phases, phase-based
Reconstruction algorithm	OSEM-PSF-TOF	TOF-OSEM
Data exported for analysis	Per-phase images and raw list-mode	Per-phase images and raw list-mode
IRB approval number	2018KT75	KHLL2023-KY125

**Table 2:** Inter-reader agreement for lesion ROI delineation across respiratory phases in the full clinical cohort.

Subgroup	Dice Similarity, Median (IQR)	Lesion-Phase Pairs
Overall	0.977 (0.907–0.984)	1642
Small lesions (<10 mm)	0.939 (0.899–0.953)	1152
Large lesions (≥10 mm)	0.981 (0.945–0.997)	490
Beijing cohort	0.969 (0.954–0.987)	1083
Kunming cohort	0.972 (0.961–0.992)	559

**Table 3:** Lesion-level SUV variability before and after motion correction, stratified by lesion size in the independent clinical test set. Values are median absolute phase-to-reference percentage differences across evaluable lesion-phase pairs, with interquartile ranges. $|∆{SUV}_{max}|$ and $|∆{SUV}_{mean}|$ were calculated relative to the fixed reference phase. Moving denotes the uncorrected moving respiratory phases before deformation-based correction and is reported as a descriptive pre-correction baseline. Lesion-size subgroups were assigned according to the diameter of the corresponding independent lesion. For registration methods, bootstrap 95% confidence intervals are reported for the median difference relative to SCAR-Net. The moving row was used only as a descriptive pre-correction baseline. Positive confidence intervals indicate higher residual SUV variability than SCAR-Net, whereas confidence intervals spanning 0 indicate no clear difference. “↓ indicates that lower values are better”.

Lesion Size	Method	$|∆{SUV}_{max}|$ (%) ↓	IQR $|∆{SUV}_{max}|$ (%) ↓	95% Bootstrap CI (vs. SCAR-Net)	$|∆{SUV}_{mean}|$ (%) ↓	IQR $|∆{SUV}_{mean}|$ (%) ↓	95% Bootstrap CI (vs. SCAR-Net)
Small	Moving	25.93	11.29	Descriptive baseline	5.58	11.68	Descriptive baseline
Small	ModeT	30.61	13.26	[+8.7, +27.9]	6.34	6.38	[+0.8, +7.4]
Small	TM	25.84	19.06	[+12.3, +27.1]	7.68	4.94	[+0.9, +6.5]
Small	VM	23.91	12.97	[+11.1, +28.4]	12.29	6.22	[+0.4, +6.0]
Small	XM	18.76	13.48	[+8.4, +26.2]	6.9	12.04	[+0.6, +6.9]
Small	GM	29.08	17.09	[+14.2, +30.6]	7.67	7.48	[+1.1, +7.2]
Small	**SCAR-Net**	6.45	7.17	Reference	4.73	4.56	Reference
Large	Moving	16.54	9.76	Descriptive baseline	5.14	4.80	Descriptive baseline
Large	ModeT	16.55	11.07	[4.29, 10.82]	4.8	5.02	[−0.41, 4.06]
Large	TM	17.54	12.14	[1.29, 10.06]	7.18	4.72	[2.38, 5.42]
Large	VM	14.6	16.43	[1.71, 9.95]	8.05	3.91	[2.28, 7.29]
Large	XM	9.33	13.26	[−2.54, 4.68]	4.56	5.79	[−1.09, 3.35]
Large	GM	14.88	12.05	[3.56, 6.65]	8.51	7.09	[1.73, 9.78]
Large	**SCAR-Net**	10.71	5.2	Reference	3.43	3.82	Reference

**Table 4:** Lesion spatial correspondence before and after motion correction in the independent clinical test set. Dice values are reported as medians across evaluable lesion-phase pairs. Lesion-size subgroups were assigned according to the diameter of the corresponding independent lesion. ΔDice denotes the paired change after correction relative to the uncorrected moving phase. Bootstrap 95% confidence intervals are reported for median ΔDice. Positive ΔDice values indicate improved lesion spatial correspondence after motion correction. “↑ indicates that higher values are better”.

Lesion Size	Method	Dice (Uncorrected Baseline) ↑	Dice (Motion-Corrected) ↑	Median ∆Dice (Corrected–Uncorrected Baseline) ↑	95% Bootstrap CI for Median ∆Dice
Small	ModeT	0.55	0.61	0.06	[0.01, 0.12]
Small	TM	0.55	0.65	0.1	[0.05, 0.23]
Small	VM	0.55	0.48	−0.07	[−0.14, 0.03]
Small	XM	0.55	0.67	0.12	[0.03, 0.31]
Small	GM	0.55	0.63	0.08	[0.01, 0.14]
Small	**SCAR-Net**	0.55	0.72	0.17	[0.06, 0.35]
Large	ModeT	0.71	0.79	0.08	[0.01, 0.36]
Large	TM	0.71	0.75	0.03	[0.01, 0.28]
Large	VM	0.71	0.82	0.11	[0.01, 0.42]
Large	XM	0.71	0.85	0.14	[0.02, 0.39]
Large	GM	0.71	0.74	0.03	[0.01, 0.21]
Large	**SCAR-Net**	0.71	0.89	0.18	[0.09, 0.38]

**Table 5:** Center-stratified lesion-level quantitative stability and spatial correspondence on the independent clinical test set. Values are medians across evaluable lesion-phase pairs. The Beijing subset included 12 patients, 24 independent lesions, and 169 lesion-phase pairs; the Kunming subset included eight patients, 19 independent lesions, and 76 lesion-phase pairs. B-spline FFD is included as a conventional deformable registration reference. Dice denotes lesion correspondence before motion correction. “↑ indicates that higher values are better, whereas ↓ indicates that lower values are better”.

Center	Method	$|∆{SUV}_{max}|$ (%) ↓	$|∆{SUV}_{mean}|$ (%) ↓	Dice (Uncorrected Baseline) ↑	Dice (Motion-Corrected) ↑
Beijing	B-spline FFD	11.57	9.65	0.60	0.69
Beijing	ModeT	24.24	5.63	0.60	0.66
Beijing	TM	22.23	7.64	0.60	0.66
Beijing	VM	20.06	12.02	0.60	0.56
Beijing	XM	15.22	5.64	0.60	0.71
Beijing	GM	23.58	7.41	0.60	0.66
Beijing	**SCAR-Net**	8.24	4.56	0.60	0.76
Kunming	B-spline FFD	21.73	11.44	0.63	0.64
Kunming	ModeT	29.51	6.24	0.63	0.68
Kunming	TM	24.92	7.31	0.63	0.72
Kunming	VM	22.53	9.02	0.63	0.64
Kunming	XM	16.71	7.08	0.63	0.76
Kunming	GM	26.31	8.87	0.63	0.67
Kunming	**SCAR-Net**	7.04	3.89	0.63	0.80

**Table 6:** Quantitative evaluation on simulated respiratory phantoms. Values are mean ± SD. Metrics summarize global image consistency, boundary discrepancy, and deformation plausibility. J≤0 denotes the fraction of voxels with a non-positive Jacobian determinant. “↑ indicates that higher values are better, whereas ↓ indicates that lower values are better”.

Datasets		SSIM(%) ↑	CC(%) ↑	NMI ↑	PSNR(dB) ↑	HD95(mm) ↓	*J* ≤ 0 ↓
CyDat	Moved	72.5 ± 0.125	71.48 ± 0.21	0.708 ± 0.098	24.3 ± 4.86	3.31 ± 0.95	—
	ModeT	85.41 ± 0.045	90.51 ± 0.036	0.722 ± 0.031	25.96 ± 1.6	2.53 ± 0.339	0.004 ± 0.0034
	TM	94.81 ± 0.028	97.71 ± 0.016	0.725 ± 0.052	32.089 ± 2.4	1.46 ± 0.43	0.0038 ± 0.0058
	VM	73.68 ± 0.13	72.29 ± 0.21	0.7026 ± 0.063	24.71 ± 4.86	3.17 ± 0.89	0.0077 ± 0.017
	GM	95.83 ± 0.02	95.94 ± 0.031	0.785 ± 0.043	32.55 ± 2.77	1.02 ± 0.086	0.0012 ± 0.0024
	**SCAR-Net**	**96.66 ± 0.018**	97.03 ± 0.021	0.7264 ± 0.059	**34.17 ± 2.88**	1.29 ± 0.34	0.0039 ± 0.0024
CaDat	Moved	91.13 ± 0.046	94.63 ± 0.043	0.9994 ± 0.0000246	27.66 ± 2.6	12.88 ± 5.42	—
	ModeT	86.79 ± 0.0092	79.21 ± 0.0086	0.8372 ± 0.0039	37.44 ± 0.53	25.4 ± 12.59	0.21 ± 0.0038
	TM	93.93 ± 0.023	97.82 ± 0.0095	0.99949 ± 2.045 × 10^−5^	30.68 ± 1.38	16.17 ± 6.23	0.0013 ± 0.001
	VM	91.57 ± 0.045	94.86 ± 0.039	0.99949 ± 1.82 × 10^−5^	27.79 ± 2.53	14.14 ± 6.72	0.009 ± 0.001
	GM	75.51 ± 0.037	86.93 ± 0.036	0.9991 ± 0.0000319	23.67 ± 0.914	23.1 ± 3.62	8.56 × 10^−7^ ± 2.1 × 10^−6^
	**SCAR-Net**	**96.78 ± 0.0068**	**99.23 ± 0.0028**	**0.9995 ± 8.47 × 10^−6^**	33.4 ± 2.81	**11.45 ± 3.12**	0.00024 ± 9.35 × 10^−5^

**Table 7:** Cross-dataset robustness testing without target-domain fine-tuning. Values are mean ± SD for image-level and deformation metrics. Small-lesion SUV metrics are reported as median absolute phase-to-reference percentage differences and were evaluated only for clinical target testing. A, B, and C denote CyDat, CaDat, and the clinical dataset, respectively. Dashes indicate metrics not evaluated for a given setting. “↑ indicates that higher values are better, whereas ↓ indicates that lower values are better”.

	Method	SSIM (%) ↑	HD95 (mm) ↓	*J* ≤ 0 ↓	$|∆{SUV}_{max}|$ (%) ↓ (Small)	$|∆{SUV}_{mean}|$ (%) ↓ (Small)
A→B	ModeT	86.91 ± 0.67	33.4 ± 10.69	0.011 ± 0.0038	—	
	TM	89.6 ± 4.22	26.65 ± 4.25	0.005 ± 0.00002	—	
	VM	89.62 ± 6	26.6 ± 4.22	0.005 ± 0.00001	—	
	XM	89.69 ± 4.6	26.6 ± 4.22	0.0092 ± 0.0012	—	
	GM	67.08 ± 2	28.07 ± 1.1	0.0052 ± 0.00003	—	
	**SCAR-Net**	94.29 ± 2.3	26.01 ± 2.67	0.0037 ± 0.00004	—	
A→C	ModeT	74.31 ± 8.5	13.33 ± 5.26	0.086 ± 0.021	4.1	14.7
	TM	88.52 ± 7.17	13.89 ± 5.3	0.005 ± 0.00001	17.04	12.19
	VM	88.52 ± 7.1	19.13 ± 7.46	0.0049 ± 0.00001	16.59	9.98
	XM	88.47 ± 7.18	14.07 ± 5.33	0.0091 ± 0.0043	14.63	8.57
	GM	77.76 ± 9.2	14.15 ± 5.87	0.005 ± 0.0002	81.28	80.64
	**SCAR-Net**	93.09 ± 1.8	17.17 ± 6.48	0.0001 ± 0.00004	4.01	8.04
B→C	ModeT	76.2 ± 6.25	18.81 ± 7.09	0.28 ± 0.027	2.5	9.8
	TM	88.52 ± 7.17	13.89 ± 5.3	0.009 ± 0.0044	26.23	14.82
	VM	88.63 ± 7	16.9 ± 7.94	0.0049 ± 0.00002	14.45	15.35
	XM	88.27 ± 6.98	13.7 ± 5.48	0.009 ± 0.0043	15.37	11.4
	GM	74.9 ± 9.5	13.95 ± 5.42	0.0052 ± 0.0002	45.28	34.23
	**SCAR-Net**	94.09 ± 2.5	16.8 ± 6.49	0.0001 ± 0.00001	2.35	8.69

**Table 8:** Computational efficiency of the selected registration methods. GPU memory, trainable parameter count, and inference time are reported for each method. Inference time is given in milliseconds per moving-reference phase pair and excludes preprocessing.

	GPU Memory (MB)	Parameter Quantity	Times (CyDat) (ms)	Times (CaDat) (ms)	Times (Clinical) (ms)
ModeT	1229.38	1,029,670	139.67	2327.78	2423.06
TransMorph	960.93	11,734,323	67.82	2381.22	2361.76
VoxelMorph	100.87	42,027	10.97	1578.78	1532.28
Xmorph	325.10	15,093,891	725.06	2685.41	2687.93
GroupM	240.15	2,884,668	2045.41	1567.81	1589.89
**SCAR-Net**	989.79	784,265	295.21	2064.62	2002.64

**Table 9:** Global ablation results on CyDat, CaDat, and the clinical dataset. Values are mean ± SD. G denotes the number of sampled group elements. Metrics summarize image consistency, boundary discrepancy, and deformation plausibility. “↑ indicates that higher values are better, whereas ↓ indicates that lower values are better”.

Dataset	Configuration	SSIM (%) ↑	CC (%) ↑	NMI ↑	PSNR (dB) ↑	HD95 (mm) ↓	*J* ≤ 0 ↓
CyDat	Backbone only	73.68 ± 0.13	72.29 ± 0.21	0.7026 ± 0.063	24.71 ± 4.86	3.17 ± 0.89	0.0077 ± 0.017
CyDat	Backbone + group-aware encoding	89.2 ± 0.036	89.65 ± 0.052	0.674 ± 0.046	28.75 ± 2.23	2.18 ± 0.66	0.02 ± 0.01
CyDat	SCAR-Net, G = 8	95.74 ± 0.022	96.13 ± 0.02	0.713 ± 0.057	33.15 ± 2.79	1.41 ± 0.23	0.0085 ± 0.002
CyDat	SCAR-Net, G = 32	96.66 ± 0.018	97.03 ± 0.021	0.7264 ± 0.059	34.17 ± 2.88	1.29 ± 0.34	0.0039 ± 0.0024
CaDat	Backbone only	91.57 ± 0.045	94.86 ± 0.039	0.99949 ± 1.82 × 10^−5^	27.79 ± 2.53	14.14 ± 6.72	0.009 ± 0.001
CaDat	Backbone + group-aware encoding	95.3 ± 0.01	98.21 ± 0.009	0.9995 ± 1.18 × 10^−5^	30.87 ± 2.02	9.85 ± 3.05	0.0081 ± 0.0005
CaDat	SCAR-Net, G = 8	95.65 ± 0.01	98.43 ± 0.004	0.9995 ± 6.5 × 10^−6^	31.96 ± 2.62	14.41 ± 5.05	0.0026 ± 0.0004
CaDat	SCAR-Net, G = 32	96.78 ± 0.0068	99.23 ± 0.0028	0.9995 ± 8.47 × 10^−6^	33.4 ± 2.81	11.45 ± 3.12	0.00024 ± 9.35 × 10^−5^
Clinical	Backbone only	88.65 ± 0.071	93.14 ± 0.045	0.399 ± 0.087	35.16 ± 4.38	13.74 ± 5.05	0.009 ± 0.004
Clinical	Backbone + group-aware encoding	93.21 ± 0.019	94.08 ± 0.021	0.4969 ± 0.096	37.63 ± 2.91	16.78 ± 5.9	0.005 ± 0.00048
Clinical	SCAR-Net, G = 8	96.74 ± 0.008	97.02 ± 0.006	0.595 ± 0.096	41.25 ± 2.91	12.43 ± 3.21	0.001 ± 0.009
Clinical	SCAR-Net, G = 32	98.35 ± 0.004	98.64 ± 0.004	0.5956 ± 0.095	43.8 ± 2.59	13.12 ± 4.23	0.0041 ± 0.002

**Table 10:** Small-lesion ablation analysis on the independent clinical test set. Values are medians across small-lesion phase pairs. SUV variability is reported as median absolute phase-to-reference percentage differences. Dice denotes lesion spatial correspondence before and after motion correction. “↑ indicates that higher values are better, whereas ↓ indicates that lower values are better”.

Configuration	$|∆{SUV}_{max}|$ (%) ↓ (Small)	$|∆{SUV}_{mean}|$ (%) ↓ (Small)	Dice (Uncorrected Baseline) ↑	Dice (Motion-Corrected) ↑
Backbone only	23.91	12.29	0.55	0.48
Backbone + group-aware encoding	17.29	8.46	0.55	0.63
SCAR-Net, G = 8	14.75	6.61	0.55	0.68
SCAR-Net, G = 32	6.45	4.73	0.55	0.72

## Data Availability

The clinical PET/CT data used in this study are not publicly available because of patient privacy protection and institutional ethics restrictions. Access to de-identified clinical data may be considered by the corresponding author upon reasonable request and subject to institutional approval and data-use agreements. The core implementation of SCAR-Net, a trained demo checkpoint, and scripts for CyDat-based inference, visualization, and evaluation are available in the project GitHub repository: https://github.com/dalas2mimi-collab/CyDat_SCAR_Net. The repository includes a representative CyDat fixed–moving image pair in raw binary .v format (fix.v and moving.v) to demonstrate the data-loading, preprocessing, phase-to-reference registration, and visualization pipeline. This representative demo pair is provided for code execution and qualitative demonstration only and does not represent the complete simulated dataset used to generate the quantitative results reported in the manuscript. The complete GATE-simulated PET datasets, full training/test split files, derived experimental measurements, and additional training configurations are available from the corresponding author upon reasonable request. The public code repository will be archived with a persistent release identifier upon publication.

## Abbreviations

The following abbreviations are used in this manuscript:

^18^F-FDG	Fluorine-18 fluorodeoxyglucose
CC	Cross-correlation
CI	Confidence interval
Dice	Dice similarity coefficient
GAB	Group-attentive block
HD95	95th percentile Hausdorff distance
IQR	Interquartile range
NCC	Normalized cross-correlation
NMI	Normalized mutual information
OSEM	Ordered-subsets expectation maximization
PET	Positron emission tomography
PSNR	Peak signal-to-noise ratio
ROI	Region of interest
SCAR-Net	Similarity-constrained adaptive respiratory registration network
Sim(3)	Three-dimensional similarity transformation group
SSIM	Structural similarity index measure
SUV	Standardized uptake value
SUV_max_	Maximum standardized uptake value
SUV_mean_	Mean standardized uptake value
